# Supplementary material for: Impact of NLRP3 Depletion on Aging-Related Metaflammation, Cognitive Function, and Social Behavior in Mice
Source: Int J Mol Sci. 2023 Nov 21;24(23):16580. doi: 10.3390/ijms242316580 (PMC10705877; doi:10.3390/ijms242316580)
Supplement: Supplementary file 1 [file ijms-24-16580-s001.zip › ijms-2583451-supplementary.pdf]

International Journal of
Molecular Sciences

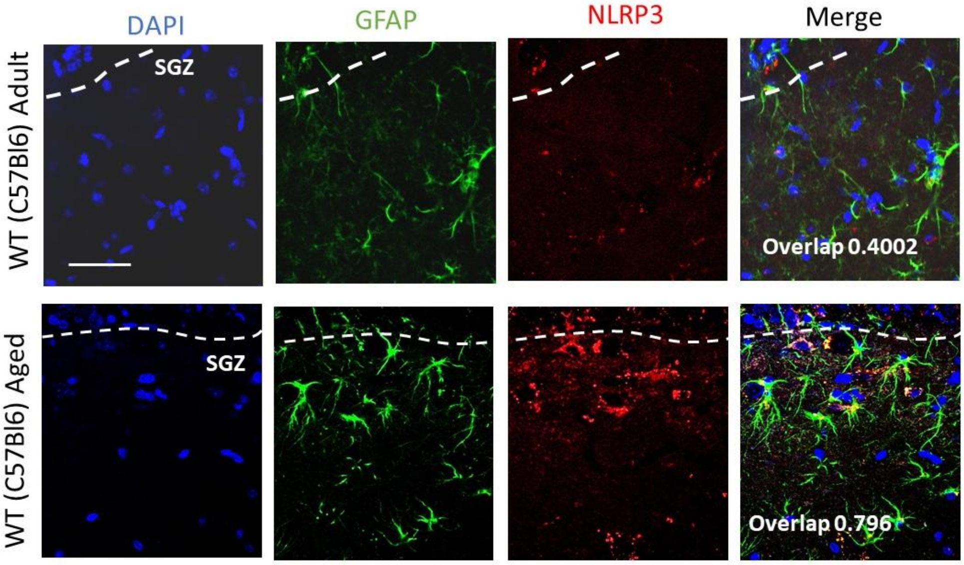

Figure S1. Representative images of the hippocampus from adult (5 months) and aged (14 months) C57Bl/6 mice stained with GFAP and NLRP3. Scale bar: 100 µm; dashed lines—subgranular zone (SGZ).

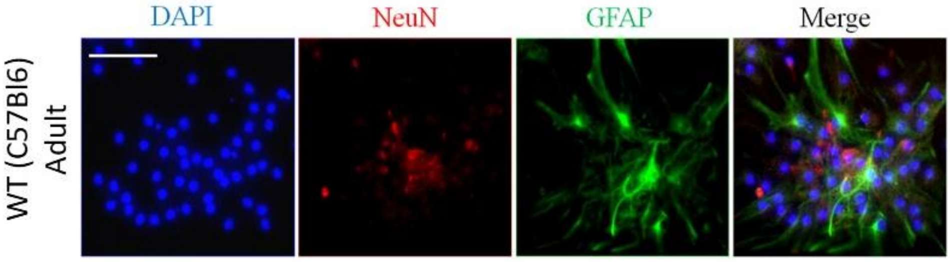

Figure S2. Representative images of the expression of the astrocyte marker GFAP and the neuronal marker NeuN in cell coculture. Scale bar: 50 µm.
